# Outcome of severe community-acquired pneumonia: the impact of comorbidities

**DOI:** 10.1186/cc11979

**Published:** 2013-03-19

**Authors:** JM Pereira, JA Paiva, F Froes, JP Baptista, J Gonçalves-Pereira

**Affiliations:** 1 Centro Hospitalar S. João, Porto, Portugal; 2 Hospital Santa Maria-CHLN, Lisboa, Portugal; 3 Hospitais da Universidade de Coimbra - CHUC, Coimbra, Portugal; 4 Hospital São Francisco Xavier, Lisboa, Portugal

## Introduction

Several comorbidities have been independently associated with both predisposition to community-acquired pneumonia and a worse outcome. The goal of this study was to evaluate the impact of comorbidities on the outcome of patients with severe community-acquired pneumonia (SCAP).

## Methods

A prospective, multicentre, observational cohort study of all patients with SCAP consecutively admitted to 14 Portuguese ICUs during a 12-month period. Several comorbidities were evaluated: congestive heart failure, cancer, chronic renal failure, chronic respiratory failure, chronic hepatic disease, alcoholism, diabetes mellitus, neurologic disease, immunosuppression, HIV infection. To evaluate the impact of comorbidities associated with hospital mortality in univariate analysis, a logistic regression analysis adjusted to other variables (clinical relevant or statistically significant in univariate analysis) was performed.

## Results

A total of 536 (14%) of the 3,766 enrolled patients had SCAP. They were mostly male (66%) with median age 59 (29 to 82) years, median SAPS II 44 (21 to 80) and total SOFA score 8 (3 to 16). Thirty-seven per cent of the cases were microbiologically documented (St. pneumoniae - 24%; Enterobacteriaceae - 20%; influenza A (H1N1) virus - 18%) and 45% had septic shock. Antibiotic combination was used in 76% of the patients and 61% received a macrolide. Median hospital length of stay was 19 (3 to 70) days and hospital mortality was 35%. Comorbidities were present in 70% of the patients. The most frequent were: diabetes mellitus (21%), chronic respiratory failure (18%) and alcoholism (15%). Median Charlson's comorbidity index (CCI) was 4 (0 to 13). In univariate analysis, the presence of at least one comorbidity (odds ratio (OR) 2.29; 95% CI 1.49 to 3.52), namely cancer (OR 3.80; 95% CI 2.14 to 6.74; *P *< 0.001), chronic renal failure (OR 3.23; 95% CI 1.53 to 6.82; *P *= 0.001), immunosuppression (OR 2.12; 95% CI 1.15 to 3.92; *P *= 0.014) and neurologic disease (OR 1.87; 95% CI 1.10 to 3.17; *P *= 0.02), increased the chances of dying in the hospital. Median CCI was also significantly higher in nonsurvivors (5 vs. 3; *P *< 0.001; OR per point 1.10 (95% CI: 1.05 to 1.15)). The only independent risk factor for hospital mortality was the presence of at least one comorbidity (OR 2.09; 95% CI 1.13 to 3.85).

## Conclusion

In SCAP, the presence of at least one comorbidity doubles the chances of dying in the hospital and is an independent risk factor for hospital mortality.

